# A Study on the Correlation Between Media Usage Frequency and Audiences’ Risk Perception, Emotion and Behavior

**DOI:** 10.3389/fpsyg.2021.822300

**Published:** 2022-01-20

**Authors:** Peng-Peng Li, Fangqi Zhong

**Affiliations:** College of Communication, National Chengchi University, Taipei City, Taiwan

**Keywords:** emergent risk events, frequency of media use, emotion, risk perception, risk control behavior

## Abstract

Whether risk events can be effectively controlled and mitigated is largely influenced by people’s perceptions of risk events and their behavioral cooperation. Therefore, this study used a web-based questionnaire (*N* = 306) to investigate the specific factors influencing people’s risk perceptions and behaviors, and included a test for the difference in the effect of positive and negative emotions of the audiences. The results show that the overall model has good explanatory power (*R*^2^ = 61%) for the behavioral variables, and (1) how people’s use of different media (especially TV and online media) significantly influenced their positive and negative emotions; (2) how people’s frequency of TV use significantly influenced their risk susceptibility and how online media use significantly influenced their risk severity (with some differences in people’s perceptions of efficacy between different media); (3) how people’s sense of efficacy for risky events is the strongest predictor of their risk control behavior; and (4) that there are different mediating effects of different emotions and risk severity and sense of efficacy between the frequency of media use and risk control behavior.

## Introduction

In December 2019, a novel coronavirus (SARS-CoV-2) emerged and was officially named by the World Health Organization (WHO) as the novel coronavirus disease COVID-19 (Coronavirus Disease-2019) in February 2020 (Zhou et al., 2020). The World Health Organization characterized the new coronavirus outbreak as a public health emergency of international concern and declared the outbreak a “Global Pandemic” on March 11, 2020. In the face of this global catastrophe, effective risk communication and management, and the promotion of risk awareness and active prevention and control behaviors among the public to reduce the risk of disease transmission, have become important issues in risk communication.

Although governments and risk management units have attempted to prevent and control the development and spread of the pandemic through various measures, such as requiring masks in public places, social distancing, providing risk protection advice, and completely blocking travel for residents (Lundgren and McMakin, 2018), effective risk assessment and management programs had to take into account the public’s perceptions and practical concerns, as risk assessment by technical experts is often based on rigorous theoretical constructs, experimental tests, and scientific evidence, whereas public risk perceptions and assessments rely more on personal values, experiences, and subjective judgments.

Public risk perceptions are heavily influenced by media coverage (Singer and Endreny, 1993; Garfin et al., 2020), whether through traditional media or online social media (Muñiz, 2011; Yoo, 2019; Huynh, 2020), or interpersonal communication (Coleman, 1993; Rogers, 2000). When faced with the risk of a life-threatening disease, the public relies on the media to obtain accurate and up-to-date risk information in order to make informed decisions about health-protective behaviors. The “Media-System Dependency Theory” (Ball-Rokeach and DeFleur, 1976) suggests that, in times of risk crisis with high uncertainty, the public increases its reliance on the media (Casero-Ripollés, 2020; Huynh, 2020; Muñiz, 2020) and tends to obtain risk assessment and risk response advice from the media they perceive as trusted (Lachlan et al., 2016).

Previous studies have generally confirmed the influence of traditional media (Loges, 1994), including television, newspapers, and magazines, on people’s risk perceptions, and in the modern era of widespread Internet access and rapid technological development, online social media have become an important channel for obtaining risk information (Karasneh et al., 2020; Malecki et al., 2020). However, it has also been suggested that, in this epidemic, people’s trust in traditional media (especially television) has increased and they are more likely to obtain risk information from traditional media (Casero-Ripollés, 2020). At the same time, people’s trust in and use of different media can significantly influence their emotions and risk perceptions (Coleman, 1993). The “risk as feeling hypothesis” suggests that people follow both cognitive (rational system) and emotional (experiential system) paths when assessing risk, and that emotions generally exert more influence on subsequent attitude formation and behavior (Loewenstein et al., 2001). For example, D’Errico and Paciello (2018, 2019) investigated the effect of people’s emotions and cognitive processes on their online comments on specific issues; and in a subsequent study, they discussed the relevance of emotions or/and cognitive processes, and behaviors, and identified the effect of negative emotions based on moral or ethical dimensions, and of self-conscious emotions based on perceptions of social (collective) norms on individuals’ pro-social behaviors (Paciello et al., 2021). In a study by Lei et al. (2014), it was suggested that the analysis of people’s (online) emotions can help to understand the diversity of cognitive responses of different individuals to specific issues and can be used to develop better marketing or communication strategies. Focusing on risk communication and management, Ning et al. (2020) examined and confirmed the importance of people’s risk perceptions, emotions, and knowledge and media messages on citizen protection behaviors during the COVID-19 outbreak through the knowledge-attitudes-practices (KAP) model. In other words, people’s emotions and their cognitive processes about specific issues can be considered as effective and attractive communication signals across social and cultural contexts, as well as business and risk management domains. In view of this, the exploration of the frequency of people’s media use under unexpected risk events and their emotional and behavioral reactions can identify promising media communication paths for effective risk communication.

In a study by Mejia et al. (2020), it was concluded that online social media and television representations of the risk of an epidemic are likely to trigger fear. Mertens et al. (2020) also concluded that fear is stronger among people who frequently use online social media to access risk information. However, although fear appeals have been shown in many studies to contribute to environmentally friendly behavior, excessive fear appeals may have negative effects (Witte, 1992; Witte et al., 2001; O’Neill and Hulme, 2009). In addition, Taha S. et al. (2014) found that during the H1N1 pandemic, anxiety was elevated when people’s perceptions of uncertainty and uncontrollability of risk information content increased. In turn, anxiety motivates people to follow preventive behaviors recommended by risk management units, such as washing hands, wearing masks, and cleaning contact surfaces (Leung et al., 2003, 2004, 2005; Quinn et al., 2009). However, most of the previous studies on risk communication and people’s perceptions and behaviors have focused on the effects of negative emotions, and the effects of positive emotions such as optimism and hope on risk-responsive behaviors have rarely been discussed. In addition to further examining the role of negative emotions in the epidemic, this study observes and discusses the influence and predictive power of positive emotions on people’s risk perceptions and behaviors during the epidemic in order to improve the model and empirical study of emotions, risk perceptions, and risk transmission.

In summary, the two main factors that influence people’s risk control behavior include emotions and risk perceptions, and the frequency of people’s media use can influence these perceptions. Therefore, this study investigated the correlation between people’s media usage frequency, emotions, and risk perceptions and behaviors using a web-based questionnaire, and then used a partial least squares approach to model the variance-based structural equation to assess the overall model’s fitness and stability, providing a reference framework for effective risk communication based on the psychological aspects of people’s emotions and risk perceptions.

## Literature Review

### Frequency of Media Use and Emotions

The Media-System Dependency Theory (MSD) (Ball-Rokeach and DeFleur, 1976) suggests that uncertain and ambiguous events or issues in society lead people to rely more on the media to resolve these ambiguous messages (Ball-Rokeach et al., 1984), and that when a major risk crisis occurs, the reliance on the media increases, and people are more likely to seek relevant information from the that than from interpersonal sources (Hirschburg et al., 1986). Similarly, Nikolaus Jackob theorized that that individuals will expose themselves to a trusted media environment (Jackob, 2010). In a study by Bangerter et al. (2012), it was confirmed that people’s media dependence positively affects media trust, which in turn can influence people’s behavioral intentions and the success of government-related policies. If people are able to obtain timely and accurate information from the medium they rely on, they will trust the medium more and continue to rely on it. Conversely, if the information provided by the medium is uncertain and ambiguous, people will trust the medium less and turn to other sources to obtain relevant information.

Before discussing the relevance of media use to emotions, it is necessary to clarify the core distinction between emotions and affect. In general, emotional responses tend to be innate, occurring automatically and quickly (Cho et al., 2014). The difference between affect and emotion is that affect is “an assessment of the overall goodness or badness of an event or issue, focusing primarily on intuitive feelings” (Slovic et al., 2004), which is the broadest definition of the term and can include moods, feelings, and emotions at the core (Damasio, 1998); whereas emotion is a series of responses to specific stimuli, such as concepts, images, and objects, from the mind or different parts of the mind to the body, resulting in changes in perception and behavior within the body (Damasio, 1998; Kittipongvises and Mino, 2013; Tsung-Jen, 2017a). Compared to the more macroscopic concept of emotions, emotions have a higher substantive meaning and can represent specific reactions of individuals to different risk issues, situations, media reports or possible behavioral consequences, such as fear, and worry (Böhm, 2003).

In the literature on listeners’ media use and their emotional responses, Ball-Rokeach and DeFleur (1976) concluded from the perspective of media dependence that listeners’ use of different media can trigger and change their emotional responses to issues. Coleman (1993) and Garfin et al. (2020) confirmed that the type and frequency of media use affects people’s psycho-emotional and physical reactions. Tracing their theoretical roots, the differential-impact hypothesis suggests that the content and function of different media (e.g., informational vs. entertainment media) exhibit different effects on the audience (Tyler and Cook, 1984; Glynn and Ostman, 1988; Coleman, 1993). As Tyler and Cook (1984) found, informational media (e.g., traditional news media) may influence individuals’ rational analyses and judgments about the risks or responses to risks faced by others or society at large, while Snyder and Rouse (1995) showed that media with rich, diverse, and lively content (e.g., entertainment media) are more likely to stimulate individuals’ emotional perceptions of what risks they will be exposed to. Holman et al. (2014), in a study examining media coverage and people’s emotional reactions in the aftermath of the Boston Marathon bombing, noted that those with high media usage experienced higher levels of “acute stress disorder,”^1^ which usually manifests as more negative emotions, such as fear, anxiety, and depression. Furthermore, Ramkissoon (2020, 2021a,b) emphasizes that family and friends or broader social network interactions increase people’s positive emotions and help people improve their sense of efficacy by observing the effectiveness of collective behaviors, which in turn leads to proper risk recognition and response. Accordingly, this study proposes:

H1:The more people receive information about the epidemic through television (H1A), online media (H1B), and interpersonal (H1C), the stronger their negative emotional responses.

H2:The frequency of people’s use of television (H2A), online media (H2B), and interpersonal (H2C) is significantly and positively correlated with their positive emotions.

### Frequency of Media Use and Risk Perception

Risk perception involves people’s subjective judgments about risks and benefits, which encompasses beliefs, attitudes, and other broader cultural values and social dispositions (UK Royal Society, 1992). In measuring people’s subjective risk perceptions, the Health Belief Model (Janz and Becker, 1984) and the Extended Parallel Process Model (Witte, 1992) of health behavior theory can be divided into three main influences: perceived susceptibility, which is the likelihood of being affected by a risky hazard; perceived seriousness, which is the severity of the impact of a risky hazard; and self-efficacy, which is an individual’s subjective behavioral judgment of his or her ability to perform or cope with a given task or situation (Bandura, 1986).

Harper et al. (2020) showed that people are more likely to engage in preventive behaviors when they perceive the risk to be serious and feel easily threatened. In a related study on efficacy, Tsung-Jen (2017b) mentioned that people assess not only their ability to act, but also the effectiveness of methods to solve problems, which is called “response efficacy.” In a subsequent discussion of the social cognitive dimension, Bandura et al. (1999) and others also referred to “collective efficacy,” the confidence of individuals knowing that collective action can solve common human problems.

The emergence of epidemics is not only caused by individuals, but also involves broader social and economic dimensions, and to some extent has connotations of collective action. Therefore, it may be more useful to consider collective efficacy along with self-efficacy and response efficacy to address high infectious risk diseases or other risk issues.

From the perspective of risk amplification by media, some scholars have suggested that the Internet can significantly enhance people’s risk perceptions and behavioral responses (Kasperson et al., 1988; Skarlatidou et al., 2012), while Morton and Duck (2001) found in their study of health risk information dissemination on risk perceptions of listeners that, for those who use traditional media to obtain risk information, traditional media directly affects their own risk perceptions, while interpersonal communication indirectly affects their own risk perceptions. Ramkissoon (2021b) mentioned that interpersonal communication directly affects people’s assessment of the severity of risk and their own sense of efficacy in coping with it. Some scholars have also argued that interpersonal communication enhances people’s risk perceptions only when the mass media is not reporting, or is balanced in its coverage of a risk issue. It can be seen that the correlation between the degree of people’s use of different media and their risk perceptions is still controversial, so:

RQ1: What is the difference between the frequency of people’s use of TV, online media, and interpersonal and their effects on risk susceptibility, severity, and efficacy?

### Emotion and Risk Perception

Empirical studies by Paul Slovic and others have shown that emotions are the main determinant of risk perception, and this pathway is considered to be the “emotional heuristic” or “risk-as-feeling” cognitive model that distinguishes it from rational risk assessment and analysis (Slovic, 1999; Finucane et al., 2000; Slovic et al., 2002; Böhm, 2003). At the same time, Lerner and Keltner (2001) argue that specific emotions can play different roles in risk perception. Also, specific emotions can have different degrees of influence on behavior (Nerb and Spada, 2001).

Experts in the field of environmental risk research have generally confirmed the positive association between negative emotions and people’s risk perceptions (e.g., Leiserowitz, 2006; Smith and Leiserowitz, 2012, 2014), such as the higher the negative emotion index, the more likely it is that the risk (climate change) is perceived to cause serious impacts, leading people to adopt relevant coping behaviors and support-related policies. Lerner et al. (2003) found that fear increases people’s perceptions of risk and harm and motivates them to act to protect themselves from potential threats, as well as confirming a positive relationship between fear, risk perceptions, and preventive behavior. In addition to fear, other negative emotions, such as anxiety and worry, have also been shown to be strongly associated with people’s risk perception and control behaviors (Leung et al., 2003, 2004, 2005; Quinn et al., 2009). However, the Extended Parallel Process Model (EPPM) suggests that the negative emotion of fear results in two opposing cognitive responses, either by acting to control the risk and harm or by refusing to act, ignoring the risk, and reducing one’s perception of fear (Witte, 1992; Witte et al., 2001). Leppin and Aro (2009) suggested that moderate levels of risk perception are beneficial for people to effectively combat epidemics and adopt preventive health behaviors, whereas high perceptions of risk of disease infection may prevent people from adopting effective preventive behaviors. It is nevertheless clear that negative emotions are mostly positively associated with people’s perceptions of risk susceptibility and severity, and significantly associated with risk prevention and control behaviors, but few studies have investigated the association between negative emotions and perceptions of efficacy, and positive emotions and risk prevention and control behaviors, so:

H3: The more negative the people’s emotions about the risk event, the higher their perception of risk susceptibility (H3A) and severity (H3B).

H4: Negative emotions are significantly and positively associated with risk control and control behaviors.

RQ2: What is the association between negative emotion and sense of efficacy?

Despite previous risk communication studies emphasizing the effect of negative emotions on risk perceptions and behaviors, the influence of positive emotions on risk perceptions and behaviors has only recently been emphasized, and the amount of research is still insufficient. Ojala’s (2012) study found that, compared to negative hope based on denial, positive hope (constructive hope) is more conducive to positive behavioral intentions in adolescents. Returning to the context of this risk event, Aslam et al. (2020) used sentiment analysis to analyze the headlines of several prominent global media outlets reporting on risk and found that, while 52% of the headlines evoked negative emotions (e.g., fear, sadness, anger), 30% of the headlines still evoked positive emotions (e.g., hope and confidence). It is clear that the media does not intend to stimulate people’s positive emotions, but positive emotions are also an important persuasion strategy. This study, however, did not explore the association between emotions, risk perceptions, and risk control behaviors.

Ramkissoon (2021a, cited Fredrickson’s (1998)) argument for positive emotions in his study on how to maintain global well-being and public health, which emphasizes that positive emotions are more effective than negative emotions in enhancing people’s psychological resilience and motivated behavioral responses to threats. However, given the lack of empirical research on the association between positive emotions and risk perceptions and behaviors, it is important to note that positive emotions are more likely to be associated with risk perceptions and behaviors, this study asks:

RQ3: Can positive emotions significantly affect the risk perception of the reader?

RQ4: Can positive emotions significantly influence the risk control behavior of listeners?

### Risk Perception and Behavior

Risk perception is a key determinant of the public’s willingness to engage in health protective behaviors. Studies of public health campaigns suggested that people’s perceptions of potential risk may influence their risk behaviors (Brewer et al., 2004); Yıldırım and Güler (2020) also suggested that people’s subjective understanding of risk in the context of new, unobservable and unpredictable risk crises (e.g., COVID-19) may influence their behavior. In another study by Bish and Michie (2010), the researchers classified the public’s risk-protective behaviors in response to pandemic diseases into three categories, namely, preventive, avoidant, and management of disease. Preventive behaviors include washing hands, covering coughs, and sneezes with tissues, cleaning touching surfaces, wearing masks, and receiving vaccinations. Avoidant behaviors include avoiding crowded places, avoiding public transportation, and following epidemic control measures. Disease management includes taking antiviral medication, seeking professional help, and using the telephone or Internet to consult about the disease.

Slovic (1987) confirmed that people are more likely to avoid catastrophic risks that may lead to immediate and widespread death and ignore slow-onset risk threats, even though such risks may cause the same or greater mortality than that caused by sudden-onset disasters. In other words, people’s perceptions of susceptibility and severity of risk increase their motivation to protect themselves, which in turn motivates them to adopt risk-averse behaviors to avoid or reduce risk (Neuwirth et al., 2000). It has been suggested that the higher people’s perceptions of their own or their friends’ vulnerability to infection, the more likely they are to adopt risk-averse behaviors, such as avoiding public places or events. It has also been suggested that the higher the perceived risk of infection to oneself or to one’s family and friends, the more likely one is to adopt risk-averse behaviors, such as avoiding public places or events and avoiding travel to countries or regions with high rates of infection (Lau et al., 2003, 2004; Blendon et al., 2004; Brug et al., 2004; Ramkissoon, 2020; Zheng et al., 2021), maintaining good personal hygiene (Tang and Wong, 2005; Wong and Tang, 2005), disinfecting homes (Lau et al., 2003; Wong and Tang, 2005; Rubin et al., 2009), wearing masks (Lau et al., 2004; Tang and Wong, 2005; Wong and Tang, 2005), strengthening the body’s immunity through diet and exercise (Tang and Wong, 2005; Wong and Tang, 2005), and vaccination (Barr et al., 2008; Ramkissoon, 2020; Qiao et al., 2021) to prevent or reduce the risk of infection.

However, earlier studies on SARS risk perception and behavior did not find significant associations between risk susceptibility and hand washing behavior (Lau et al., 2007) and other preventive behaviors (Quah and Hin-Peng, 2004; Leung et al., 2005). In addition, people actively seeking drug prophylaxis, as well as counsel from experts, family or friends when dealing with infectious health risks, can reflect more diversity of perceived needs and behavioral motivations at the psychological level of the reader, Interpersonal communication can also influence the effectiveness of risk management to some extent (Ramkissoon, 2020, 2021a,b) and can be considered as an important observation and an area that needs to be urgently explored under COVID-19 risk events (2021b). This study defines this type of behavior as “disease management” and uses the research model to observe the representativeness and effectiveness of this type of behavior. In summary, this study proposes that:

H5:People’s risk susceptibility (H3A), severity (H3B), and efficacy (H3C) were significantly and positively associated with their risk prevention and control behaviors.

**FIGURE 1:**
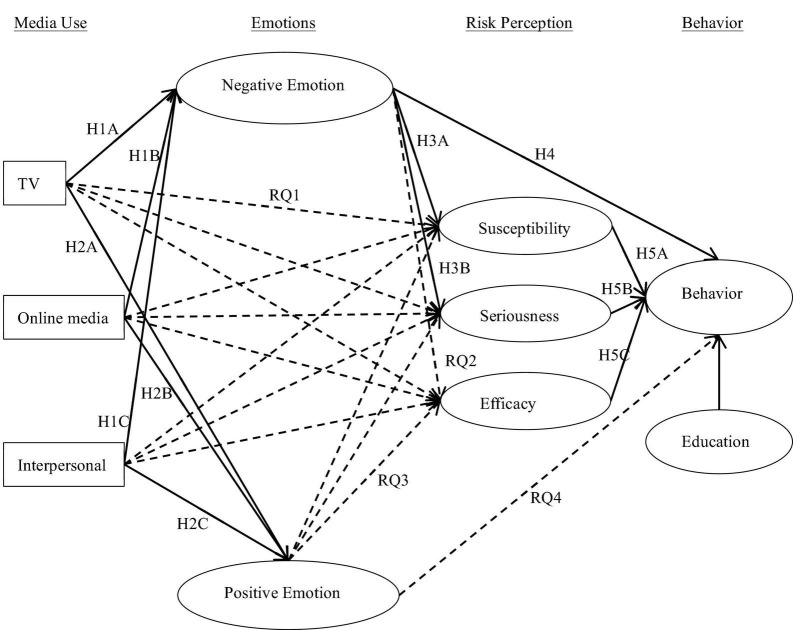
Initial study model.

Combining the above research hypotheses and questions, the initial model of this study is shown in Figure 1.

## Materials and Methods

### Survey Instrument and Sample Structure

Because the web-based questionnaire format can collect a large number of samples in a short period of time and has the advantages of low cost, visualization, and ease of subsequent research analysis (Fowler, 2002; Vaughn and Turner, 2016), it is gradually becoming one of the important tools for survey research at this stage. However, web-based questionnaires have disadvantages, such as under-representation, lack of sampling frame, and voluntary sampling, which become key issues to be addressed when choosing this method for data collection. Couper (2000), Witte and Allen (2000), and Dillman (2011) suggest that the data collection process should reflect the heterogeneity of the sample, the sample size should be increased and compared with the existing database, and the data collected should be adjusted by using statistical weighting, while avoiding excessive inferences when conducting research analysis. Inferences can effectively improve the reliability and validity of the questionnaire.

In addition, in terms of research tools, web-based questionnaire systems have become more and more functional in recent years and can largely meet the needs of researchers for different types (e.g., surveys, experiments) of research. At present, the more popular web-based questionnaire systems in the academic field are SurveyMonkey (United States), Questionnaire Star (Mainland), and SurveyCake (Taiwan). These three questionnaire systems all have the core functions of design, collection, and analysis, however, users can pay SurveyMonkey to specify the group to be tested, but its test samples are mainly people in Europe and the United States, and the subscription fee is high. Questionnaire Star is a simplified Chinese interface, its functions and models are rich with a low subscription fee. SurveyCake emerged in the Taiwan market, and is mainly used in academic research in Taiwan. The Chinese version of SurveyCake has been widely used to survey people’s perceptions of issues and behavioral intentions in mainland China because of its relatively low subscription cost and mature development. Therefore, SurveyCake was used as the online survey tool in this study.

The questionnaire was firstly published on Weibo, WeChat friends circle, Xiaohongshu, and other online media with a large number of users, followed by contacting community management committees, college and university teachers and disseminating the questionnaire link, inviting people aged 18 and above in mainland China to fill in the questionnaire voluntarily. The system prevents each IP address from filling in the questionnaire more than once (through the duplicate IP deletion function of Questionnaire Star). The survey was conducted starting on December 20, 2020 and ended on January 6, 2021 with a total of 306 valid questionnaires collected.

Among the valid sample, 172 (56.2%) were male and 134 (43.8%) were female. The age distribution was mainly concentrated between 18 and 55 years old and relatively evenly distributed, ranging from 26 to 30 years old (17.7%), 20 to 25 years old (16.9%), 31 to 35 years old (15.6%), 36 to 40 years old (14.2%), 41to 45 years old (13.5%). In terms of respondents’ marital status, the majority of respondents were married, with 135 (44.1%), followed by 93 (30.4%) who were cohabiting, 65 (21.2%) who were unmarried, 7 (2.3%) whose spouses had died, and 6 (2.0%) who were divorced or separated. The education degrees of the respondents were bachelor (103, 33.7%), master (79, 25.8%), high school (57, 18.6%), junior high school (31, 10.1%), doctoral (29, 9.5%), and others (7, 2.3%). In terms of sample structure, the gender, age, and marital status are very similar to the recent demographic data in mainland China, which represents the representativeness and inferred universality of the online sample. Only “education” differs from the census database. Lion et al. (2002) and Clayton and Myers (2015) pointed out that education is one of the internal factors that influence people’s risk-responsive behavior, so this study set it as a control variable to observe its effect on the dependent variable.

### Definition and Measurement of Variables

#### Frequency of Media Use Under Emergent Risk Events

The frequency of media use usually represents the exposure time and the degree of involvement and dependence of the reader on different media (Greenwald and Leavitt, 1984; Chaffee and Schleuder, 1986; de Vreese and Neijens, 2016). Mei-Ling and Yie-Jing’s (2011) measurement of this variable specifically asks, “to what extent did you obtain information related to the outbreak during the New Coronary Pneumonia outbreak from the following media?” The options were (1) television, (2) online media, including WeChat, Weibo, and QQ, and (3) interpersonal, including family and friends, neighbors, and peers (colleagues), using a five-point scale [from 1 = never, 2 = rarely, 3 = sometimes (2–3 times a week), 4 = often (4–6 times a week), to 5 = almost every day].

#### Emotions

Emotions are a series of responses to specific stimuli, such as concepts, images, and objects, that move from the mind or different parts of the mind to the body, resulting in changes in perception and behavior within the body (Damasio, 1998; Kittipongvises and Mino, 2013; Tsung-Jen, 2017a). The scale used to measure the emotions of listeners in this study was based on Russell (1980, 2003), Russell and Barrett’s (1999) “circumplex model,” Scherer’s (2005) “emotional dimensions scale,” and Plutchik’s (1984) “three-dimensional model of emotion structure,” and was revised with reference to Briesemeister et al.’s (2011) emotion glossary, Mikels et al.’s (2005) and Stevenson et al.’s (2007) emotion measures, and the specificity of this study’s topic. A five-point scale (1 = strongly disagree, 2 = disagree, 3 = unsure, 4 = agree, 5 = strongly agree) was used to ask: “How much do you agree with the following emotional adjectives in describing your emotional state during the New Coronary Pneumonia outbreak?” There were five adjectives representing positive emotions in the scale, namely optimistic, hopeful, confident, calm, and reassuring, and five adjectives representing negative emotions, namely fearful, depressed, scared, upset, and worried. Based on the overall model pretest results, adjectives with low factor loadings (<0.6 =) were removed (Hair et al., 1992), and finally, the observed variables of negative emotions were selected based on factor loadings > 0.6: fearful (0.82), worried (0.80), and anxious (0.78); the observed variables of positive emotion adjectives included: hopeful (0.88), confident (0.84).

#### Risk Perception

Referring to Rogers and Prentice-Dunn (1997) Health Belief Model (Janz and Becker, 1984) and the Extended Parallel Process Model (Witte, 1992) of protective motivation theory and health behavior theory, three main potential variables were split to measure risk susceptibility perceptions, risk susceptibility perceptions, and efficacy (Frewer et al., 2002).

Perceptions of risk susceptibility (i.e., likelihood of being at risk of harm): (1) To what extent do you think you are susceptible to COVID-19, (2) To what extent do you think you can avoid getting COVID-19, and (3) To what extent do you think you are a potential factor in spreading COVID-19 (five-point scale: 1 = not at all, 2 = not, 3 = not sure/don’t know, 4 = probably, 5 = to a large extent).

Sense of efficacy (includes confidence in individual and collective ability to cope with risk and the effectiveness of risk response programs): (1) Do you think you can do simple things to reduce the threat of NCCV infection; (2) Do you think government measures to prevent NCCV infection (e.g., wearing masks, washing hands, keeping social distance, etc.) can reduce the spread of NCCV and the chance of infection; (3) Do you think that the measures provided by the government to prevent the spread of neoplasmosis (such as wearing a mask, washing hands regularly, keeping social distance, etc.) can reduce the chance of transmission and infection of neoplasmosis (5-point scale: 1 = not at all, 2 = not at all, 3 = not sure/don’t know, 4 = yes, 5 = to a large extent).

#### Risk Control Behavior

Referring to Bish and Michie’s (2010) analysis, this study classified behavioral response intentions into three categories: risk prevention, risk avoidance, and disease management, and asked participants on a five-point scale (1 = definitely not, 2 = not, 3 = not sure, 4 = probably, 5 = definitely) how likely they would be to adopt the following behaviors during a new coronary pneumonia outbreak, including:

Risk prevention behaviors: (1) wearing a mask outside; (2) washing hands regularly; (3) covering up with a tissue when coughing or sneezing; (4) cleaning the surfaces of touching objects.
Risk avoidance behaviors: (1) avoid going out; (2) if you must go out, avoid taking public transportation; (3) if you must go out, avoid crowded places; (4) avoid buying food for delivery.
Disease management behaviors: (1) taking antiviral medications to prevent viral infection; (2) using the telephone or Internet to consult professionals about symptoms of new coronary pneumonia or prevention advice; (3) proactively discussing disease related information with friends, relatives or others through the Internet, social media or face-to-face.

In order to simplify the research model as much as possible, these 11 behavioral items were first analyzed by principal component factor analysis, and then were extracted by Bartlett’s test of sphericity using the eigenvalue greater than one rule. The total variance was 77.13% explained by the three factors (i.e., the observed constructs of risk prevention, avoidance, and disease management behavior intention). Also, the factor loadings of the 11 items of the scale were all greater than 0.7, which has high convergent validity (Hair et al., 1992). Therefore, in the formal analysis of this study, the scores of the three types of behavioral items were summed, with higher scores indicating higher willingness to respond to the behavior in question.

### Non-response Bias

Referring to Armstrong and Overton’s (1977) and Shiau and Luo’s (2012) test procedures for non-response bias, this study examined the issue of non-response bias by comparing the gender and age of early (*N* = 187) and late (*N* = 119) participants. The chi-square test for early and late participants showed no significant difference in gender or age (*p* > 0.05). Therefore, the possibility of non-response bias was ruled out.

### Common Method Bias

In this study, the questionnaire was designed using the “Questionnaire Star” software and the data were distributed and collected through an online questionnaire. In order to ensure the validity of the follow-up analysis, on the one hand, the questionnaire was designed in accordance with previous research theories and implementation methods. The main purpose of the study was described to the participants, ethics and precautions were informed, the questions were filled in anonymously, and the questions of each variable were randomly ordered to minimize the influence of CMB on the results of the study. On the other hand, we used a *post hoc* test (Lindell and Whitney, 2001) in the later screening of the valid sample. Following the procedure of Hultman et al. (2009), we isolated the observed variables with a minor positive correlation (0.015) and then calculated the CMB-adjusted correlation between all latent variables and found that the difference between the original and adjusted correlation coefficients was very small (*r* ≤ 0.015) and the significant correlation of the variables did not change. At the same time, the chi-square test of fit showed no significant results (X^2^ = 17, *p* > 0.05), so the CMB had no significant effect on the results and inferences of this study.

### Partial Lease Square-Structural Equation Modeling Method

Structural equation modeling (SEM) allows us to assess the correlation and theoretical connection of one or more independent variables with one or more dependent variables in terms of discrete or continuous variables (do Nascimento and da Silva Macedo, 2016). Its advantage lies mainly in the ability to effectively analyze the causal relationships between multiple variables (Williams et al., 2009; Durdyev et al., 2018). There are two standard approaches to SEM, namely Covariance Base (CB-SEM), based on covariance, often using LISREL, EQS, and AMOS software, and partial lease square (PLS), which analyzes the principal component structure of variables. In the past decades, CB-SEM has been considered the main way to analyze the complex relationship between observed and latent variables, while PLS-SEM has become more popular in recent years in social science research. Both have unique advantages in terms of analytical sample structure, objectivity, and model fitness, but the use of PLS-SEM is more appropriate when the research goal is to develop a theory with a small sample size and a more complex model (Hair et al., 2019; Khan et al., 2019; Shiau et al., 2019; Wen-Long, 2020). On the contrary, if the study aims to verify or confirm theoretical connections, CB-SEM is the most appropriate method (do Nascimento and da Silva Macedo, 2016; Peng et al., 2019).

PLS-SEM is more suitable for this study than CB-SEM for the following reasons: (1) this study is an exploratory study aimed at testing and developing existing theories and research results; (2) the analytical results are used to predict variable correlations; (3) there are more variables and the overall model is more complex; (4) the effective sample size collected is smaller; and (5) the sample distribution lacks normality (Gefen et al., 2011; Hair et al., 2019; Khan et al., 2019; Shiau et al., 2019; Wen-Long, 2020). Therefore, in this study, we ran SmartPLS 3 to test the reliability and validity of this structural model by plotting the model and validating the factor analysis. Table 1 shows that all the factors in this study have a good convergent validity with factor loadings > 0.6 (Hair et al., 1992; Wixom and Watson, 2001) and *p* < 0.01 (Bock et al., 2005). Each construct has an AVE indicator > 0.05 (Chin et al., 2003). This means that the factors in this study met the stability criteria.

**TABLE 1:** Scale properties of structure model.

Construct	Item	Mean	Standard deviation	Error loading	Standardized item loading	T-statistic
TV	TV	3.76	0.74	0.37	0.88	29.17***
Online media	Online media	3.91	0.69	0.29	0.86	32.33***
Interpersonal	Interpersonal	3.85	0.67	0.35	0.87	37.64***
Positive emotion	Promising	3.55	0.84	0.32	0.88	42.91***
	Confident	3.64	0.77	0.30	0.84	37.26***
Negative emotion	Fearful	3.99	0.61	0.29	0.82	25.77***
	Worried	3.97	0.66	0.26	0.80	39.97***
	Anxious	3.88	0.73	0.22	0.78	43.10***
Susceptibility	Sus1	3.47	0.80	0.24	0.78	39.95***
	Sus2	3.61	0.78	0.19	0.80	47.83***
	Sus3	3.42	0.81	0.27	0.88	53.21***
Seriousness	Ser1	3.88	0.68	0.24	0.83	48.75***
	Ser2	3.86	0.67	0.18	0.88	21.45***
	Ser3	3.75	0.73	0.19	0.86	51.00***
Efficacy	Se	3.39	0.81	0.34	0.81	44.17***
	Re	3.44	0.81	0.33	0.79	29.93***
	Ce	3.78	0.76	0.27	0.80	42.24***
Behavior	preventive	3.62	0.79	0.30	0.89	59.25***
	avoidant	3.87	0.70	0.24	0.87	45.69***
	management	3.73	0.79	0.33	0.85	36.27***

*p < 0.05; **p < 0.01; ***p < 0.001.

Next, this study examined the correlation and extracted average variance (AVE) of the potential variables to confirm the discriminant validity of the observed items (Gefen et al., 2000). The results showed the discriminant validity of the potential variables for subsequent analysis and inference (see Table 2).

**TABLE 2:** Scale properties and correlations.

Construct	Number of items	Reliability	AVE	Factor correlations									
				TV	OM	IP	PE	NE	Sus	Ser	Ef	Be	Ed
TV	1	0.93	0.74	1.0									
OM	1	0.95	0.72	–0.01	1.0								
IP	1	0.90	0.77	0.05	0.12	1.0							
PE	2	0.88	0.70	0.62	0.28	0.14	1.0						
NE	3	0.90	0.78	0.33	0.44	0.37	0.13	1.0					
Sus	3	0.94	0.81	0.53	0.18	0.16	0.09	0.08	1.0				
Ser	3	0.92	0.83	0.47	0.50	0.28	0.04	0.14	0.16	1.0			
Ef	3	0.91	0.80	0.66	0.73	0.22	0.31	0.17	–0.18	–0.37	1.0		
Be	3	0.95	0.75	0.22	0.25	0.17	0.28	0.27	0.36	0.29	0.34	1.0	
Ed	6	0.93	0.86	0.70	0.69	0.66	0.55	0.25	0.44	0.36	0.29	0.61	1.0

*OM, Online medi; IP, Interpersonal; PE, Positive Emotion; NE, Negative Emotion; Sus, Susceptibility; Ser, Seriousness; Ef, Efficacy; Be, Behavior; Ed, Education.*

## Structural Modeling Analysis

**FIGURE 2:**
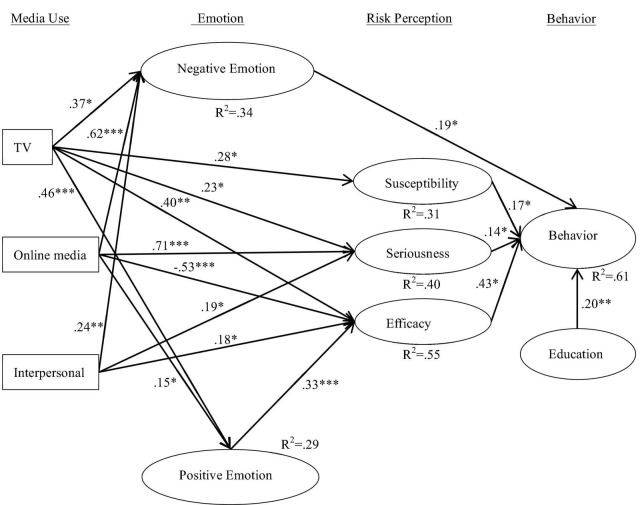
Results of the structure model. *N* = 306, *p < 0.05; ^**^p < 0.01; ^***^p < 0.001.

After testing the reliability and validity of the initial research model, this study proceeded to perform path analysis and correlation testing of the overall model using PLS. The path coefficients and significance of each research hypothesis in the structural model were verified by a bootstrap resampling method (1,000 resamples), and the results are shown in Figure 2.

From the results of the analysis, the model explained 34% of the variance in negative emotions, 29% of the variance in positive emotions, 31% of the variance in susceptibility, 40% of the variance in severity, 55% of the variance in efficacy, and 61% of the variance in behavior.

First, in terms of the correlation between people’s media usage frequency and their emotions: (1) people’s frequency of online media usage was significantly positively correlated with their negative emotions (β = 0.62, *p* < 0.001), i.e., the more people tended to obtain epidemic-related information from online media, the stronger their negative emotions were (H1B holds); (2) its significance exceeded the significant positive correlation between TV usage frequency and their negative emotions (β = 0.37, *p* < 0.05), i.e., the more people tended to obtain epidemic-related information from TV media, the stronger their negative emotions (H1A holds); (3) the significant positive correlation between frequency of interpersonal network use and negative emotions (β = 0.24, *p* < 0.05), i.e., the more people tend to obtain epidemic-related information from interpersonal media, the stronger their negative emotions (H1C holds). In terms of the correlation between people’s frequency of use of different media and their positive emotions; and (4) the more often people obtained risk information through television (β = 0.46, *p* < 0.001) and online media (β = 0.15, *p* < 0.05), the stronger their positive emotions were (H2A, H2B), but the frequency of interpersonal use was not significantly correlated with positive emotions (H2C did not hold).

Secondly, from the statistical results of people’s frequency of use of different media and their risk perceptions, although people’s frequency of use of online media was not significantly correlated with their risk susceptibility, it was significantly correlated with people’s risk severity and sense of efficacy, and its significance exceeded the effects of television and interpersonal on people’s risk susceptibility and sense of efficacy (RQ1), specifically: (1) the higher the frequency of people’s use of television, the higher their risk susceptibility (β = 0.28, *p* < 0.05), severity (β = 0.23, *p* < 0.05), and efficacy (β = 0.40, *p* < 0.01); (2) the more frequently people used online media, the higher their risk severity (β = 0.71, *p* < 0.001) and lower their efficacy (β = −0.53, *p* < 0.001); and (3) the more frequently people used interpersonal higher frequency, the higher their risk severity (β = 0.19, *p* < 0.05) and sense of efficacy (β = 0.18, *p* < 0.05).

Again, in terms of the association between people’s emotions and risk perceptions and risk control behaviors, the statistical results showed that (1) people’s negative emotional responses to the risk event were not significantly associated with their risk susceptibility (β = 0.09, *p* > 0.05) and severity (β = 0.07, *p* > 0.05) (H3 does not hold), nor with their sense of efficacy (β = 0.10, *p* > 0.05) [RQ2 (RQ2)]; (2) people’s positive emotional response to the risk event was significantly and positively correlated with their sense of efficacy (β = 0.33, *p* < 0.001), i.e., the stronger people’s positive emotions were, the higher their confidence in themselves, their government, and the group to fight the risk, but positive emotions were not significantly correlated with their risk susceptibility and severity (RQ3); (3) people’s negative emotions were weakly and significantly positively correlated with their risk control behavior (β = 0.19, *p* < 0.05); and (4) people’s negative emotions were weakly and positively correlated with their risk control behavior (RQ4) (β = 0.19, *p* < 0.05), i.e., moderate negative emotions help to stimulate positive risk prevention behavior (H4 holds). There was no direct significant relationship between people’s positive emotions and their risk control behavior (β = 0.04, *p* > 0.05) (RQ4).

Finally, in terms of the association between risk perception and risk control behavior, the statistical results confirmed that people’s risk susceptibility (β = 0.17, *p* < 0.05), severity (β = 0.14, *p* < 0.05), and efficacy (β = 0.43, *p* < 0.05) were significantly and positively associated with their risk control behavior, i.e., the more people perceived the risk as serious, their vulnerability to infection and injury, and their confidence in risk control, the more positive their risk prevention behavior was.

In addition, this study also examined the indirect pathway of media frequency on risk-causal behavior, i.e., whether emotions and risk perceptions mediated the effect of media frequency on risk-causal behavior. The results of the analysis, controlling for educational attainment variables, showed that (1) negative emotions mediated the positive effect of TV (95% CI: 0.42–0.51, *P* < 0.001) and online media (95% CI:0.58–0.73, *P* < 0.001) usage frequency on behavior; (2) positive emotions mediated the positive effect of TV usage frequency on behavior (95% CI: 0.19–0.32, *P* < 0.01); (3) positive emotions mediated the positive effect of TV usage frequency on behavior. *P* < 0.01); (4) sense of efficacy mediated the positive effect of TV usage frequency on behavior (95% CI: 0.29–0.36, *P* < 0.01); (5) risk severity mediated the positive effect of interpersonal usage frequency on risk prevention behavior (95% CI: 0.31–0.45, *P* < 0.05); and (6) online media mediated the positive effect on behavior through both risk severity and sense of efficacy was positively influenced (95% CI: 0.17–0.31, *P* < 0.01).

## Discussion

This study investigated the factors that influence people to adopt positive risk prevention behaviors in the context of sudden risk events (COVID-19), and whether the frequency of using different media is significantly related to their emotions (positive and negative), risk perceptions (susceptibility, seriousness, and efficacy), as well as the mediating effect the paths between media frequency and risk-responsive behaviors may have.

Our findings indicate, first, that the degree (frequency) of people’s use of different media in this COVID-19 risk event significantly affected their negative emotions of fear, worry, and anxiety, and the influence of online media on their negative emotions exceeded that of interpersonal and television, while positive emotions were mainly influenced by people’s use of television. In contrast, online media showed a weak correlation with positive emotions. Possible explanations include:

(1) When a sudden risk event occurs, scientists and traditional media (e.g., television) have difficulty in reporting accurate risk information in a timely manner, because there is not yet sufficient scientific data to confirm the appearance of risk, its destructive power, and effective preventive measures (Henrion and Fischhoff, 1986), and people may become anxious and worried because of the uncertainty and ambiguity of risk information. When scientists and media have conclusive evidence of risk hazards and countermeasures, they still cannot change their emotions or even doubt the accuracy and credibility of the information (Nyhan and Reifler, 2010), and so turn to online media and people to obtain relevant information. However, because there are many unverified messages on the Internet, people’s negative emotions intensify and spread.

(2) People may be influenced by their education level, risk knowledge, risk experience, and values to trust the risk information and response advice provided by the official media on TV, and then rationally analyze risks and make rational behavioral decisions. This finding is in line with Tyler and Cook (1984) analysis of the association between information media (traditional media) and people’s rational analysis and assessment of risk, which suggests that traditional media are still effective in guiding people to respond rationally to risk. The trust of the population in traditional media (especially TV) has increased and they are more likely to obtain risk information from traditional media. In addition, due to the aggregation of information preferences of different people by big data, people who perceive things more rationally or objectively on a daily basis are more likely to see information content that is consistent with their perceived preferences when risks occur, such as scientific analysis of risks and how to prevent infection. However, these people are still a minority in society as a whole, so online media is less influential than television in terms of positive sentiment. In terms of the association between interpersonal communication and emotion, this study did not reach the same conclusion as Ramkissoon (2020, 2021a,b)) that social network interaction (interpersonal communication) would enhance people’s positive emotion.

Second, people’s perceptions of risk susceptibility are mainly influenced by their frequency of using TV, while their perceptions of risk seriousness are mainly influenced by their frequency of using online media, followed by TV and interpersonal. Their sense of efficacy varies depending on their frequency of using different media. This conclusion supports Ramkissoon’s (2021b) comments on the importance of interpersonal communication, which significantly and positively affects people’s perceptions of risk severity and efficacy. People’s frequency of using TV and interpersonal enhances their sense of efficacy, while the higher the frequency of using online media, the lower their sense of efficacy. This finding suggests that people’s frequency of media use is indeed significantly related to their risk perceptions (Morton and Duck, 2001), but that the use and trust of different media have different effects on their risk susceptibility, seriousness, and efficacy.

Our third finding confirmed that people’s perceived efficacy of the COVID-19 risk event was the strongest predictor of their risk-response behavior, followed by negative emotion, risk susceptibility, and seriousness in that order. This finding is consistent with the findings of numerous studies focusing on the fact that people are more active in risk prevention behaviors when they perceive the risk to be serious and feel easily threatened (Harper et al., 2020), and when they perceive that individual (Tsung-Jen, 2017a) and collective action (Bandura et al., 1999; Ramkissoon, 2021b) can effectively counteract risk harm (Ramkissoon and Smith, 2014; Ramkissoon, 2020).

As for mediating effects, our fourth finding shows that different emotions and risk seriousness and efficacy have different mediating effects between the frequency of media use and risk control behavior. Emotions and risk seriousness and efficacy are important mediating variables in people’s processing of risk information. As shown in the two-path model proposed by Stúrmer and Simon (2004) and the findings of Tsung-Jen (2017a), people will act accordingly because of their negative emotions, due to the perceived relevance of risk to them, and will also enhance their sense of efficacy and behavioral intentions with the risk coping information obtained from the media.

The model explained 34% of the variance in negative emotions; 28% of the variance in positive emotions; 31% of the variance in susceptibility; 40% of the variance in severity; 55% of the variance in efficacy; and 61% of the variance in risk prevention and control behaviors. According to Cohen (1988) and Falk and Miller (1992), R Square should be equal to or greater than 0.10, which means that the variance explanation of the endogenous structure is sufficient, and the larger the value, the stronger the explanatory power of the antecedent variable for that variable. It can be seen that the structural model in this study has strong explanatory power for people’s risk prevention behavior. Therefore, this study advises that the media should pay more attention to the presentation of risk information in future risk communication, fully consider the possible effects of risk information on people’s emotions and risk perceptions, and avoid using strong negative emotional appeals (e.g., panic) and language that can be easily misunderstood (e.g., a word may have multiple meanings, some words may be used in a wide variety of ways, some words may change their meaning when combined with other words, or the meaning of a word may change), or ambiguous information (e.g., a word may mean different things to different people, such as “significant,” Tucker and Ferson, 2008), to give people confidence in their ability to cope with risk and thus to adopt positive and effective risk prevention behaviors.

As Lundgren and McMakin (2018) suggest, demonstrating the ability of government and related entities to protect the public from risk or to provide sufficient empathy to the public in the dissemination of risk information and processes is the most critical element to enhance organizational trust and effective risk management. The significant impact of traditional media on the public’s ability to correctly perceive and analyze risks and to make appropriate behavioral decisions should not be overlooked. On the one hand, considering the changing media habits of the public, government or relevant risk management units should make good use of online media, such as disseminating risk-related information in a timely manner, understanding the psychological state of the public and their general concerns and communicating with them effectively, and improving the presentation of risk information to the general public to facilitate their clear understanding and perception of risks.

On the other hand, due to the high accessibility and low threshold of online media, people are easily influenced by “news” that is mixed with the real and the fake, and so may spread the unchecked information to their personal social networks and larger media platforms, resulting in an increase of negative emotions, such as collective fear, worry, and anxiety (Taha S. A. et al., 2014; Jones et al., 2017). They may overestimate certain threat factors that are actually less risky or underestimate certain factors that are actually more risky, resulting in one-sided or erroneous risk perceptions (Slovic et al., 1980), and even disordered or overly aggressive risk responses and violent emotional outbursts, such as hearing rumors that spraying alcohol on masks can improve their protective ability. This is why government has to take a more active role in communication and management. This means that in the process of risk communication and management, government should improve the transparency of information and the speed of updating information, prevent people from speculating and spreading vague or uninformed risk information as much as possible, establish fact-checking platforms and mechanisms to stop the vicious spread of fake news in online media and among people, and strengthen the cultivation of media literacy among people in the field of education, so as to effectively manage risks and promote a rational society.

Previous health risk communication studies have mostly focused on protective motivation and health belief models, exploring the effects of perceived severity, perceived susceptibility, perceived benefits, and perceived barriers on risk prevention and treatment behaviors of listeners. However, COVID-19 is a global risk event that involves both environmental and health risks, which are not only related to health practices and health at the individual level, but also to the collective interests of society. This requires a more diversified exploration of the psychological factors that influence the behavior of listeners, as discussed in Loewenstein et al.’s (2001) risk-as-feelings hypothesis, which suggests that people will follow both cognitive and emotional paths to form specific attitudes and make behavioral decisions, which affirms the influence of emotions on attitudes and behaviors. However, subsequent research on the influence of emotions on risk-responsive behavior has focused on negative emotions (especially fear) and has failed to identify in detail the effects of which negative emotions and positive emotions influence behavior.

Among the many studies on negative emotions and behaviors, the Extended Parallel Process model (EPPM) suggests that listeners’ processing of risk information can be divided into threat assessment and effectiveness assessment (Witte, 1992; Witte et al., 2001). The threat assessment includes the listener’s assessment of the severity and susceptibility of the risk, or the relevance of the risk, while the efficacy assessment includes the assessment of one’s own and the group’s ability to cope with the risk, and the effectiveness of risk advice. Combined with the Health Belief Model and the EPPM, this study classifies risk perception into three important dimensions: susceptibility, severity, and efficacy, and analyzes the differential effects of different (positive/negative) emotions and risk perception dimensions on the frequency of media use and the risk prevention and control behavior paths of the listeners. The results of this study are not only applicable to the field of health risk communication, but also have an important reference value in the field of environmental risk communication.

However, the types of media in modern society are very diverse, and it is difficult to clearly distinguish which media are traditional media or online media. It is suggested that, in the future, we can increase the number of observed variables of online media (e.g., official web pages, online search engines, outdoor online media, short video platforms, etc.) and dialogue with the results of this study to further enhance the explanatory validity and generalizability of this study’s model. At the same time, in addition to the core variables discussed in this study, the factors influencing individuals’ risk-responsive behavior should not neglect the guiding or moderating effects of individual values, risk knowledge, risk experience, social norms, and other socio-demographic variables (e.g., gender, age, residence, etc.) on their behavior (see Mary and Wildavsky, 1982; Morgan et al., 1985; UK Royal Society, 1992; Peters and Slovic, 2000; Edelstein, 2002; Slovic, 2016).

As suggested by Ramkissoon (2020, 2021a,b), place attachment and habitual behavior are also important factors influencing people’s risk perceptions and behaviors, and have an important impact on people’s well-being and health. Although this study has controlled for the influence of educational attainment on behavior, given the parsimonious nature of the overall research model, other factors influencing behavior have not been discussed comprehensively, and it is suggested that future research could add or change the control variables and add moderating variables to observe and test whether the explanatory power of this study’s model and findings have changed and what differences exist.

In addition, considering the urgent need to maintain people’s health and enhance human well-being in the context of modern risk society and globalization (2021b), future research may focus on the ecological environment and people’s psychological recovery after the epidemic, and how to enhance people’s happiness and well-being, such as building (Ramkissoon, 2021a) and promoting (Ramkissoon, 2020) emotional connections between individuals and activity sites, and the establishment and promotion of ecotourism to repair negative emotional stress (Majeed and Ramkissoon, 2020), and to compare the psychological and behavioral effects of different social institutions and cultural contexts on people’s risk coping.

## Data Availability Statement

The original contributions presented in the study are included in the article/supplementary material, further inquiries can be directed to the corresponding author/s.

## Author Contributions

P-PL: conceptualization, methodology, data curation, and writing-original draft preparation. FZ: supervision and writing- reviewing and proofing. Both authors contributed to the article and approved the submitted version.

## Conflict of Interest

The authors declare that the research was conducted in the absence of any commercial or financial relationships that could be construed as a potential conflict of interest.

## Publisher’s Note

All claims expressed in this article are solely those of the authors and do not necessarily represent those of their affiliated organizations, or those of the publisher, the editors and the reviewers. Any product that may be evaluated in this article, or claim that may be made by its manufacturer, is not guaranteed or endorsed by the publisher.

## Footnotes

1 Acute stress disorder is defined as a reaction of fear, helplessness, or terror that reaches pathological levels after an individual experiences a traumatic stressful event (Department of Psychiatry, National Taiwan University School of Medicine Hospital, 2015).
